# High Metastaticgastric and Breast Cancer Cells Consume Oleic Acid in an AMPK Dependent Manner

**DOI:** 10.1371/journal.pone.0097330

**Published:** 2014-05-13

**Authors:** Shuai Li, Ti Zhou, Cen Li, Zhiyu Dai, Di Che, Yachao Yao, Lei Li, Jianxing Ma, Xia Yang, Guoquan Gao

**Affiliations:** 1 Department of Biochemistry, Zhongshan School of Medicine, SunYat-sen University, Guangzhou, China; 2 Department of Physiology, University of Oklahoma, Health Sciences Center, Oklahoma City, Oklahoma, United States of America; 3 Key Laboratory of Functional Molecules from Marine Microorganisms (Sun Yat-sen University), Department of Education of Guangdong Province, Guangzhou, China; 4 China Key Laboratory of Tropical Disease Control (SunYat-sen University), Ministry of Education, Guangzhou, China; Wayne State University, United States of America

## Abstract

Gastric cancer and breast cancer have a clear tendency toward metastasis and invasion to the microenvironment predominantly composed of adipocytes. Oleic acid is an abundant monounsaturated fatty acid that releases from adipocytes and impinges on different energy metabolism responses. The effect and underlying mechanisms of oleic acid on highly metastatic cancer cells are not completely understood. We reported that AMP-activated protein kinase (AMPK) was obviously activated in highly aggressive carcinoma cell lines treated by oleic acid, including gastric carcinoma HGC-27 and breast carcinoma MDA-MB-231 cell lines. AMPK enhanced the rates of fatty acid oxidation and ATP production and thus significantly promoted cancer growth and migration under serum deprivation. Inactivation of AMPK attenuated these activities of oleic acid. Oleic acid inhibited cancer cell growth and survival in low metastatic carcinoma cells, such as gastric carcinoma SGC7901 and breast carcinoma MCF-7 cell lines. Pharmacological activation of AMPK rescued the cell viability by maintained ATP levels by increasing fatty acid β-oxidation. These results indicate that highly metastatic carcinoma cells could consume oleic acid to maintain malignancy in an AMPK-dependent manner. Our findings demonstrate the important contribution of fatty acid oxidation to cancer cell function.

## Introduction

Epidemiological and animal studies have demonstrated an association between fatty acids (FA) or obesity and the cancer tumourigenesis and metastasis [1], [2]. Advanced gastric cancer and breast cancer have a clear tendency towards metastasis and invasion to the microenvironment, which is predominantly composed of adipocytes [3], [4]. Oleic acid is the most common monounsaturated FA in human adipocytes and other tissues [5]–[7]. Relatively little is known regarding whether highly metastatic gastric and breast cancer cells could adapt to the highly fatty acid culture and gain a survival/growth advantage by metabolic transformation to utilise oleic acid as an energy source.

Studies from recent decades have reported accumulating evidence of metabolic reorganisation during cancer development in various tumour types [8]. One of the first biochemical hallmarks of cancer cells to be identified were the marked changes in metabolism [9]. Tumour cells gain a survival/growth advantage by adapting their metabolism to respond to environmental stress, a process known as metabolic transformation. The best-known aspect of metabolic transformation is the Warburg effect [10]. Recently, several lines of evidence implicate fatty acid oxidation (FAO) as an important contributor to metabolic transformation [11]–[15], indicating that fatty acid metabolism might contribute to cancer cell function. With most cancer researchers focusing on glycolysis, glutaminolysis and fatty acid synthesis, the relevance of fatty acid oxidation (FAO) to cancer cell function has not been carefully examined. In particular, little is known regarding the biochemical pathways by which oleic acid influences tumour progression.

One of the fundamental requirements of all cells is the balancing of ATP consumption and generation [16]. Tumour cells typically undergo metabolic transformation modulated by AMPK [17]–[20]. AMPK is a highly conserved sensor of cellular energy status that exists in the form of heterotrimeric complexes containing a catalytic α-subunit combined with regulatory β and γ-subunits [21], [22]. In response to energy depletion, AMPK activation promotes metabolic changes to maintain cell proliferation and survival by directly phosphorylating rate-limiting enzymes in metabolic pathways, modifying the signal transduction cascades and gene expression [23]. FAO induction downstream from AMPK activation might be a survival or growth strategy employed by some cancer cells subjected to metabolic stress [20].

It has been reported recently that omental adipocytes promote homing, migration and invasion of ovarian cancer cells [24]. Adipocyte-ovarian cancer cell coculture led to the direct transfer of lipids from adipocytes to ovarian cancer cells and promoted tumour growth, suggesting that there is a link in cancer cells between the adaptation to consume exogenous energy and the ability to migrate. It is unknown whether oleic acid provides an energy source for other highly metastatic carcinoma cells; if so, there is a question regarding the basis of molecular mechanism.

It is essential to elucidate the molecular mechanisms by which oleic acid regulates the malignant behaviour of high metastatic cancer cells. To clarify the unknown problems, we based our work on the premise that assessing the influence and mechanism of oleic acid on cancer cells would provide a better understanding of fatty acid metabolism and the molecular mechanisms present in gastric and breast cancer cells.

## Materials and Methods

### Chemical

Fatty acid-free BSA was obtained from Wako Pure Chemical Industries, Ltd., and oleic acid, AICAR and Compound C were purchased from Sigma.

### Cancer Cell Lines

The HGC-27, AGS, SGC7901, BGC823 human cancer cell lines were obtained from the Cell Bank of the Chinese Academy of Science. The MDA-MB-231 and MCF-7 cells were obtained from the American Type Culture Collection (ATCC). The MCF-7 and MDA-MB-231 cells were maintained in DMEM supplemented with 10% heat-inactivated foetal calf serum (FCS), and the HGC-27, AGS, SGC7901 and BGC823 cells were maintained in RPMI 1640 supplemented with 10% FCS. The cells were grown in monolayer cultures at 37°C in a humidified atmosphere of 95% air and 5% CO_2_. When BSA-bound fatty acids were added to the serum-free culture medium, the final concentration of BSA was adjusted to 0.5%.

### Cell Viability Assay

The cells in 48-well plates at a density of 10,000 cells per well were treated with different stressors. The cell viability was measured using the 3-[4,5-dimethylthiazol-2-yl]-2,5-dephenyl tetrazolium bromide (MTT) assay (Roche Co.), according to the manufacturer’s protocol.

### BrdU Incorporation Assay

The cell proliferation was determined by measuring the BrdU incorporation using a BrdU incorporation assay (Roche Molecular Biochemicals), according to the manufacturer’s instructions. Briefly, 5,000 cells/well seeded in a 96-well plate were pulse-labelled for 2 h with 10-uM BrdU. The cells were incubated for 30 min with a diluted, peroxidase-conjugated anti-BrdU antibody. The absorbance values were measured at 450 nm using an ELISA reader (Bio-Rad iMark).

### Migration Assay

The cell migration assays were performed using Transwell chambers (8-µm pore size polycarbonate membrane, Corning). A total of 50 K cells were plated into the insert in 200 µl serum-free medium containing BSA or 400 µM BSA-bound oleic acid and allowed to migrate from the upper compartment to the lower compartment toward a 15% FBS gradient for the indicated time period. After the migration, the non-migratory cells on the upper membrane surface were removed by scrubbing, and the membrane was fixed in buffered 4% paraformaldehyde and stained with 0.1% crystal violet at room temperature. The migrated cells were then enumerated. The migration values were expressed as the average number of migrated cells per microscopic field over six fields per assay from three independent membrane experiments.

### Western Blotting

The cells were harvested and lysed for the total protein extraction. The protein concentration was determined using a Bio-Rad DC protein assay kit (Bio-Rad Laboratories) according to the manufacturer’s protocol. The aliquots of equal amounts of protein from the cell lysate were subjected to western blot analysis. The antibodies used in this study include those specific for phospho-Thr172-AMPKa (Cell Signaling Technology, Danvers, MA, #4188s), AMPKa (CST, #2532s), phospho-Ser96-ACC (CST, #3661S), ACC (CST, #3676s), ATGL (CST, #2138S), caspase9 (CST,#9502s), caspase3 (CST, #9662s), CPT1a (Proteintech group, 15184-1-AP), MCAD (Proteintech group, 55210-1-AP), GAPDH(Santa Cruz, sc-365062) and β-actin (Sigma,MO, USA,A5441). The densitometry was performed using ImageJ software (developed by Wayne Rasband, National Institutes of Health, Bethesda, MD; available at http://rsb.info.nih.gov/ij/index.html) and normalised by the GAPDH or β-actin levels.

### siRNA Transfection

The AMPKα1 siRNA and a nonspecific siRNA (control) were purchased from RiboBio. According to the manufacturer’s instructions, the transfections were performed at approximately 60% confluency using Lipofectamine 2000 (Invitrogen). For each transfection reaction, 20 nM AMPKα1 siRNA or control siRNA was used for the preparation of the siRNA-transfection complexes at room temperature for 20 min. The transfections were performed in 0.5-(12-well plate) or 1.5-mL (6-well plate) serum-free medium for 8 hr. After incubation, the transfection complexes were removed and replaced with their corresponding media. The transfection efficiency (80% –90%) was determined by western blotting analysis. The cells were used for western blotting analysis and cell viability 24–72 hr after transfection.

### Oil Red O Staining and Triglyceride Assay

The lipid droplets were stained by Oil red O. A stock solution was prepared in 2-propanol (0.3%), and a working solution was freshly prepared by diluting the stock solution with water (3∶2). After fixation, the cells were washed twice in PBS and stained with Oil red O and hematoxylin for 15 min and 1 min, respectively. The cells were washed with PBS, and images were obtained under a light contrast microscope. The intracellular triglycerides were assayed using a triglyceride assay kit (GPO-POD; Applygen Technologies, Inc., Beijing, China) according to the manufacturer’s recommended protocol.

### Determination of Oxygen Consumption

Five million cells were resuspended in 1 ml of fresh warm medium pre-equilibrated with 21% oxygen and placed in a sealed respiration chamber equipped with a thermostat control, micro stirring device and Clark-type oxygen electrode disc (Oxytherm, Hansatech Instrument, Cambridge, UK). The oxygen content in the cell suspension medium was constantly monitored for 10 min, and the oxygen consumption rate was recorded.

### ATP Assays

The intracellular level of ATP was measured with an ATPlite assay kit (Beyotime Biotech). The cells were incubated in a serum-free medium with or without 400 µM of OA for 48 h, washed with PBS three times, lysed in ATP extraction buffer and centrifuged in 4°C. ATP was measured by luminometric methods using commercially available luciferin/luciferase reagents on a luminometer (TD-20/20; Turner Designs) according to the manufacturer’s instructions. The data were normalised to total protein.

### Statistical Analysis

The data are presented as the means ± SD. SPSS 13.0 software was used for the statistical evaluation using one-way ANOVA for comparison of more than two groups, and LSD-t test, Dunnett’s T3 or Tamhane’s T2 were used for the multiple comparisons. Student’s t test was used for the comparison of the two groups. P values of less than 0.05 were considered significant.

## Results

### The Opposite Effects of Oleic Acid on the Cell Viability of Various Cancer Cell Lines

**Figure 1:**
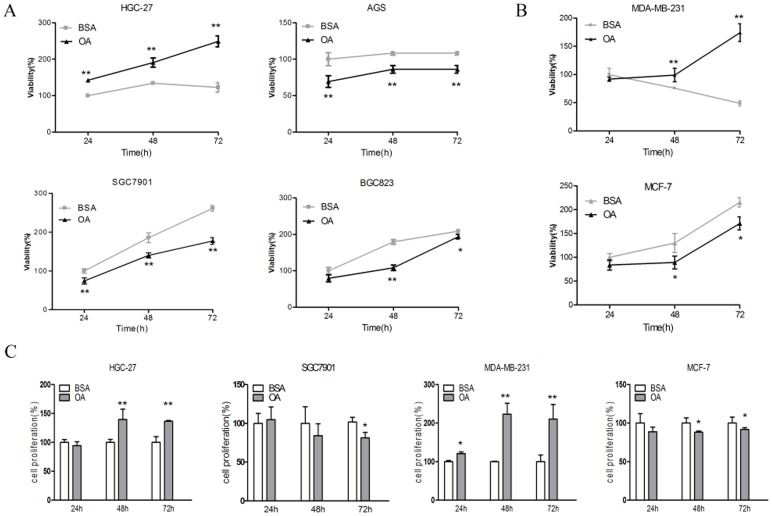
Effects of oleic acid on cell survival in various human cancer cell lines. Human gastric cancer cell lines (A) and breast cancer cell lines (B) were starved for 12 hours and then incubated with 0.5% BSA as the control or 400 µM BSA-bound oleic acid for the indicated time period (hours). The cell viability was determined by an MTT assay and expressed as a percentage of the control cells. The values are the mean ± SD, n = 3; **p<0.01 vs. BSA at the indicated time points. Student’s t test. (C) The cells were incubated with 0.5% BSA or 400-µM BSA-bound oleic acid for the indicated time points, and the cell proliferation was examined using BrdU ELISA. The values are the mean ± SD, n = 3; *p<0.05 and **p<0.01 for OA compared with BSA.Student’s t test.

To better clarify the role of oleic acid in tumour growth, we examined the effects of oleic acid on the cell viability of human gastric carcinoma cell lines and breast cancer cell lines, respectively. As shown in Fig. 1A, 400 µM of BSA-bound oleic acid stimulated obvious cell viability in HGC-27 cells after 24, 48, and 72 hours of exposure. A decrease in cell viability was observed in the AGS, SGC7901 and BGC823 cells treated with OA. In the MDA-MB-231 cells, OA had an effect on cell viability similar to that of the HGC-27 cells, more profoundly with the 72-hours treatment. We observed an inhibitory effect of OA on cell viability in the MCF-7 cells (Fig. 1B).

For a better assessment of the reason for the cell viability change induced by OA, we first determined the cell proliferation using BrdU ELISA. The assessment shows that cell proliferation was significantly higher in the OA groups compared with the control group in the HGC-27 and MDA-MB-231 cells; OA exhibited a slight inhibition of cell proliferation in the SGC7901 and MCF-7 cells (Fig. 1C). The Hoechst staining and flow cytometry analysis indicated that OA afforded protection against apoptosis in the HGC-27 and MDA-MB-231 cells whereas it induced apoptosis in the SGC7901 cells and MCF-7 cells. Mechanically, the level of cleaved caspase3 and cleaved caspase9 were changed in the OA-treated cells compared to that in the control group, suggesting that mitochondrial apoptotic pathway might be involved in this progress (Figure S1).

### Different Effects of Oleic Acid on Cell Migration

**Figure 2:**
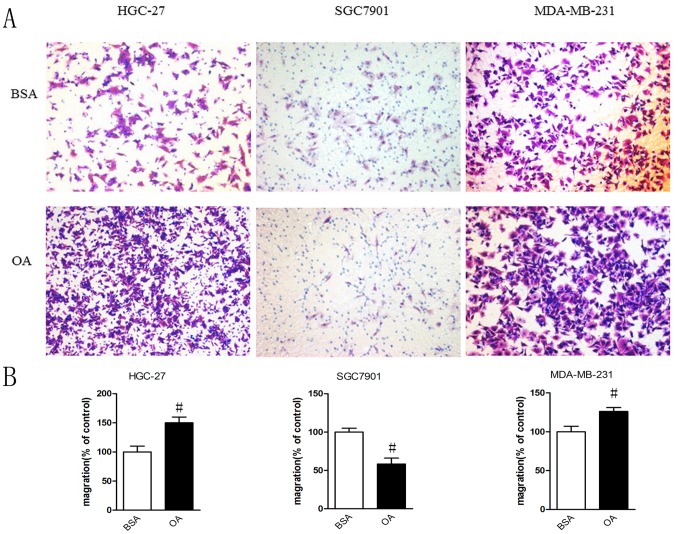
Effects of oleic acid on cell migration of gastric carcinoma cells and breast cancer cells. Cells treated with 0.5% BSA or 400 µM BSA-bound oleic acid were allowed to migrate from upper compartment to lower compartment toward a 15% FBS gradient for 12 hours in HGC-27 and SGC7901 cells and 6 hours in MDA-MB-231 cells. (A) After then the migrated cells were fixed, stained, and photographed (magnification 100 x) and (B) the migrated cells were then enumerated and normalised to the control, #p<0.05 for OA compared with BSA.Student’s t test.

To further determine the action of OA on cell migration, we used Transwell chamber assays to gauge the migratory ability of the cells treated with OA. The cells were plated into the insert in a serum-free medium containing BSA or 400 µM of BSA-bound oleic acid and allowed to migrate from the upper compartment to the lower compartment toward a 15% FBS gradient. OA prompted an obvious increase in the migration of the HGC-27 and MDA-MB-231 cells, whereas this process was reduced in the SGC7901 cells (Figure 2).

### Effects of Oleic Acid on the Activation of AMPK Signalling and Downstream Enzymes

Exogenous OA transported into the cells is converted into acyl-CoA, which is esterified with glycerol to yield inert triacylglycerols (TGs) or β-oxidation [25], [26].

**Figure 3:**
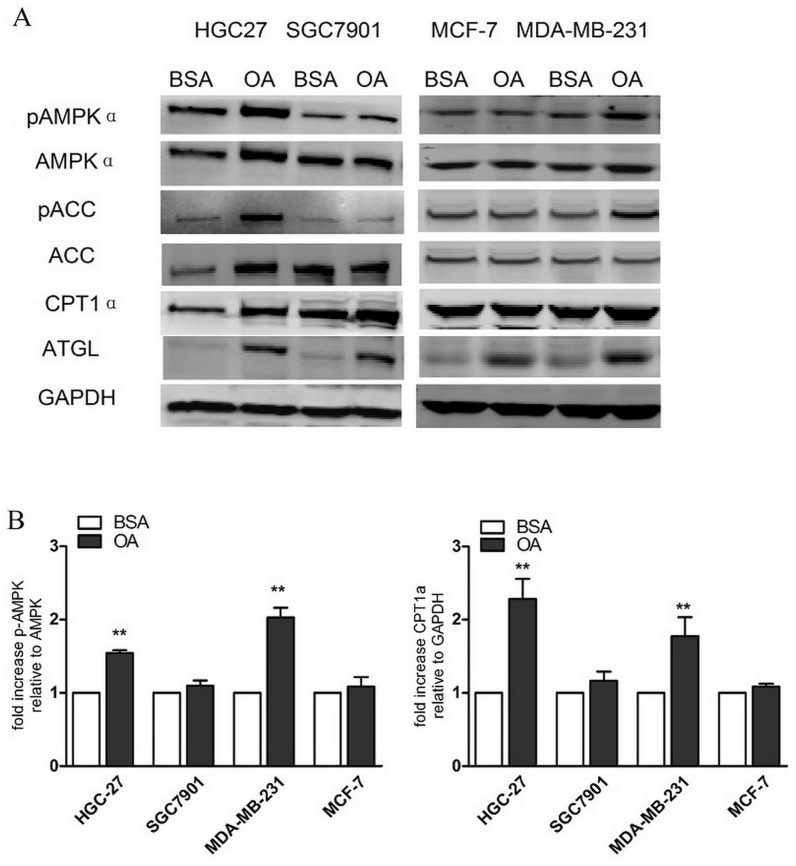
The AMPK pathway was selectively activated in various human cancer cells treated with oleic acid. (A) The cells were incubated with 0.5% BSA or 400 µM of BSA-bound oleic acid for 48 hours. The levels of phosphorylated AMPK (Thr172), the total AMPK, pACC (Ser79), the total ACC, CPT1α and ATGL were determined by western blot analysis using 50 µg of the total proteins from each sample. (B) The Quantification of Protein expression by densitometry from three independent experiments normalised to GAPDH. The values are expressed as the percent of control cells, given as the mean ± SD, n = 3; **p<0.01 for OA compared with BSA.Student’s t test.

Based on these findings, we hypothesised that the disparity in the cellular biological function treated by OA between the high metastatic cancer cells (HGC-27, and MDA-MB-231) and low metastatic cancer cells (SGC7901, MCF-7) is ascribed to the difference in the ability of intracellular TG lipolysis. We detected the expression of adipose triglyceride lipase (ATGL), which catalyses the initial step of lipolysis, converting TGs to diacylglycerols (DGs). ATGL was completely stimulated in all the cells treated by OA compared to the control cells (Fig. 3A), demonstrating that lipolysis was not the distinction separating the high metastatic cancer cells from the low metastatic cancer cells.

AMPK, a fuel sensor, plays an important role in regulating fatty acid β-oxidation and is involved in the different effects OA has on cell function. We performed a comprehensive analysis of the expression of AMPK and a downstream gene by western blotting in various cancer cells. Phosphorylation of AMPKα was selectively up regulated by OA in the HGC-27 and MDA-MB-231 cells, and there was no obvious change in the SGC7901 and MCF-7 cells (Fig. 3A & B).

Carnitine palmitoyl transferase 1(CPT1α), which catalyses the transport of long-chain fatty acids (LCFAs) into mitochondria for β-oxidation, is highly inhibited by the malonyl CoA that is catalysed in mitochondria by acetyl-CoA carboxylase (ACC). AMPK could phosphorylate and inactivate ACC, thus the ACC-dependent malonyl CoA levels fall, releasing the inhibition of CPT1α, which facilitates the mitochondrial entry of the LCFAs for β-oxidation [21], [27]. As shown in Fig. 3A, OA significantly increased the phosphorylation of ACC in the HGC-27 cells and mildly affected the ACC in the MDA-MB-231 cells. The expression of CPT1α was up regulated consistently by OA in those two cell lines (Fig. 3A & B).

Consistently, the oxygen consumption rate of the MDA-MB-231 cells in the medium containing OA was significantly higher compared to that of the cells grown in the control medium. The oxygen consumption rate of the MCF-7 cells grown in the medium containing OA did not differ significantly from that of the control cells (Figure S2). These results suggested that AMPK activation participated in the utilisation of OA through β-oxidation and maintained a metabolic advantage.

### The Different Actions of OA on Cellular Behaviour are AMPK Dependent

**Figure 4:**
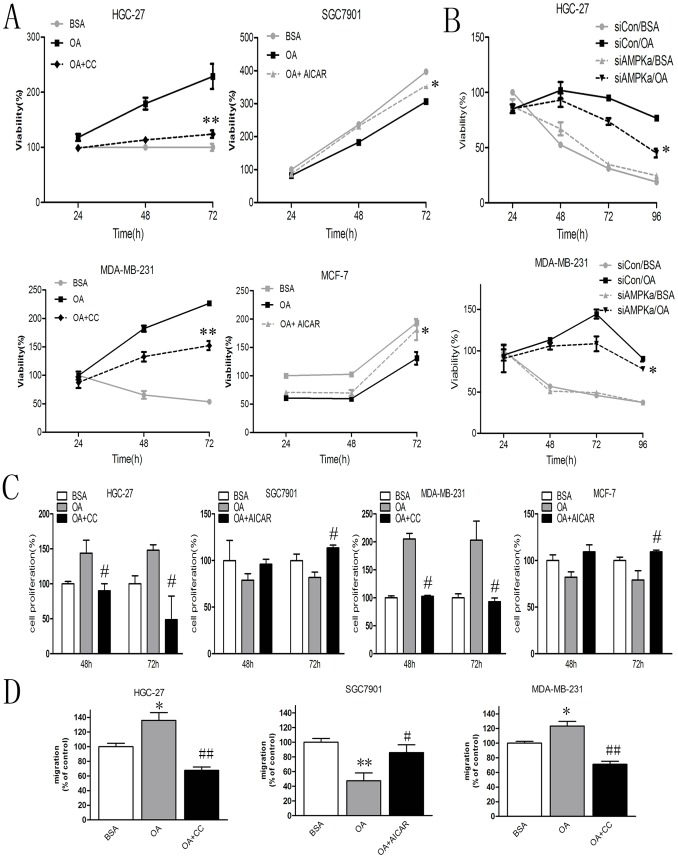
AMPK activation facilitates cancer cell growth and survival in the presence of oleic acid. (A) The cells were cultured with 0.5% BSA or 400 µM of BSA-bound oleic acid with 5 µM of Compound C or with 100 µM of AICAR for 24, 48, of 72 hours. The cell viability was determined using a MTT assayat the indicated time points. The values are the mean ± SD, n = 3; *p<0.05 and **p<0.01 for OA+Compound C or OA+AICAR compared with OA.one-way ANOVA followed by Fisher’s LSD test. (B) The cells transfected with control-siRNA or AMPKα1-siRNA were treated with 0.5% BSA or 400 µM of BSA-bound oleic acid for 24, 48, 72, of 96 hours. The cell viability was determined using an MTT assay and expressed as the percentage of the control cells at the indicated time points. The values are the mean ± SD, n = 3; *p<0.05 for OA+ AMPKα1-siRNA compared with OA+control-siRNA.one-way ANOVA followed by Fisher’s LSD test.(C) The cells were cultured with 0.5% BSA or 400 µM of BSA-bound oleic acid with 5 µM of Compound C or with 100 µM of AICAR for 48 or 72 hours. The cell proliferation was examined using BrdU ELISA at the indicated time points. The values are the mean ± SD, n = 3; # p<0.05 for OA+Compound C or OA+AICAR compared with OA. one-way ANOVA followed by LSD test. (D) The statistical comparison of the percentage of the control cells in the cell-migration ability (the cells treated with 0.5% BSA or 400 µM of BSA-bound oleic acid with 5 µM of Compound C or with 100 µM of AICAR were allowed to migrate for 12 hours in the HGC-27 and SGC7901 cells and for 6 hours in the MDA-MB-231 cells.). All of the statistical analysis values represent the mean of three experiments (the means ± SD, n = 3, *p<0.05 and **p<0.01 for OA compared with BSA; #p<0.05 and ##p<0.01 for OA+Compound C or OA+AICAR compared with OA. one-way ANOVA followed by LSD test.

To further confirm that AMPK activation was involved in the utilisation of OA for growth, survival and migration in the cancer cell lines, 5-aminoimidazole-4-carboxamide-1-β-D-ribofuranoside (AICAR) and Compound C were used. As shown in Figure 4A, the addition of Compound C to the HGC-27 and MDA-MB-231 cells suppressed the facilitation of growth by OA. The treatment with AICAR restrained the growth inhibition by OA in the SGC7901 and MCF-7 cells.

We designed siRNA specific to the AMPK catalytic α1-subunit to reduce the protein levels because AMPK is activated by phosphorylation of Thr172 on the catalytic α-subunit. The western blot assay confirmed an obvious reduction in the protein level in the MAD-MB-231 cells (Figure S3). As depicted in Figure 4B, consistent with the exposure to Compound C, silencing of the AMPK catalytic α-subunit by treatment with OA caused a reduction of cell viability after 72 hours.

There was a question regarding whether the alteration of cell proliferation and migration relied on AMPK activation in the cells treated by OA. As shown in Figures 4 C&D, Compound C elicited a 1.0 to 1.5-fold decrease in cell proliferation and migration in the OA-treated HGC-27 and MAD-MB-231 cells, whereas the reduction of cell proliferation and migration in the OA-treated SGC7901 and MCF-7 cells was compensated for by AICAR. Collectively, these data suggested that AMPK activation is critical for the cancer cell survival and growth induced by OA.

### The Activation of AMPK is a Key Factor to Utilising OA and Providing ATP for Cancer Cell Growth and Survival

**Figure 5:**
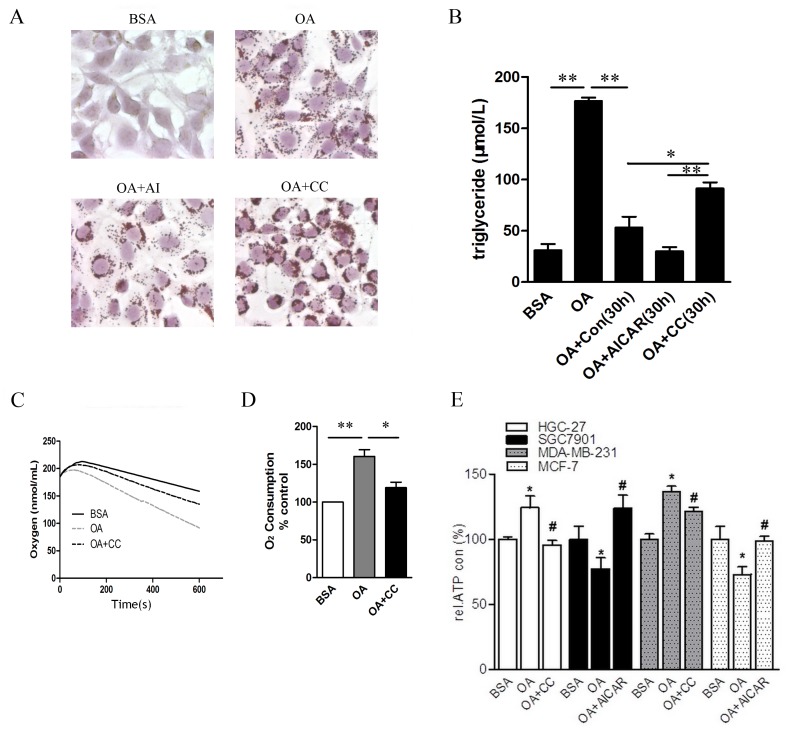
AMPK is pivotal to utilise OA and provide ATP for cancer cell growth and survival. (A) The MDA-MB-231 cells were cultured with 0.5% BSA or 400 µM of BSA-bound oleic acid with 5 µM of Compound C or with 100 µM of AICAR for 48 h. The cells were stained with Oil red O with 400× magnification. (B-D) The MDA-MB-231 cells were pretreated with 400 µM of oleic acid for 6 h and refreshed with medium containing 5 µM of Compound C or with 100 µM of AICAR for another 30 h. (B) The cells were digested, and the intracellular triglycerides were assayed using a triglyceride assay kit, normalised to the protein content.The data are presented as the mean ± SD, n = 3, *p<0.05, **p<0.01. (C) The oxygen content in the cell suspension medium was constantly monitored for 10 min in the different groups. (D) The oxygen consumption was normalised against the number of viable cells being measured. The data are presented as the mean ± SD, n = 3, *p<0.05, **p<0.01. (E) Various cancer cells were cultured with 0.5% BSA or 400 µM of BSA-bound oleic acid with 5 µM of Compound C or with 100 µM of AICAR for 48 h. The ATP level was determined with a commercial assay and normalised to the protein content. The data are presented as the mean ± SD, n = 3, *p<0.05 for OA compared with BSA; #p<0.05 for OA+Compound C or OA+AICAR compared with OA. Multiple groups were compared using one-way ANOVA followed by Fisher’s LSD test.

We assessed whether AMPK has a function in regulating the lipid accumulation induced by OA. Consistent with other observations [28], the lipid droplet material stained by Oil red O increased in the OA-treated MDA-MB-231 (Fig. 5A), HGC-27 and SGC7901 cells compared to the control cells (Figure S4), suggesting that cancer cells are able to esterify oleate for storage as TG. The lipid droplets in the OA-treated MDA-MB-231 cells were diffused with small granules, indicating that the TG stored in lipid droplets was simultaneously hydrolysed and provided with energy. Consistent with the Oil red O staining, the cellular triglyceride content assay indicated that OA induced TG accumulation significantly for the initial 6 hours, which was consumed during the next 30 hours in incubation with fresh medium in the absence of OA (OA+Con). Compound C attenuated the TG consumption compared to the control group (OA+Con), as shown in Figure 5B. This finding demonstrated that the inhibition of AMPK signalling impeded the utilisation of OA and fatty acid metabolism in an obvious manner.

To further elucidate the mitochondrial respiratory chain activity, we measured the oxygen consumption rate in the cells with different treatments. This result indicated that OA stimulated the oxygen consumption whereas Compound C reduced the oxygen consumption in the MDA-MB-231 cells, suggesting that AMPK might participate in the intracellular fatty acids flux into mitochondria for β-oxidation (Fig. 5 C&D).

We determined whether AMPK activation is directly attributable to the ATP production influenced by the OA stress in our cell models. The intracellular levels of ATP in the various treated groups of different cell lines were measured (Fig. 5E). As shown in figure 5E, the inhibition of the AMPK signalling by Compound C attenuated the increase of ATP production in the HGC-27 and MDA-MB-231 cells treated with OA, whereas the AICAR compensated for the ATP level by activating the AMPK signalling in the SGC7901 and MCF-7 cells.

These data support our hypothesis that the activation state of AMPK is a switch in the cells to respond to OA by mediating fatty acid metabolism.

### AMPK Regulated the Key Enzymes Involved in β-oxidation in OA- treated-high Metastatic Cancer Cells

**Figure 6:**
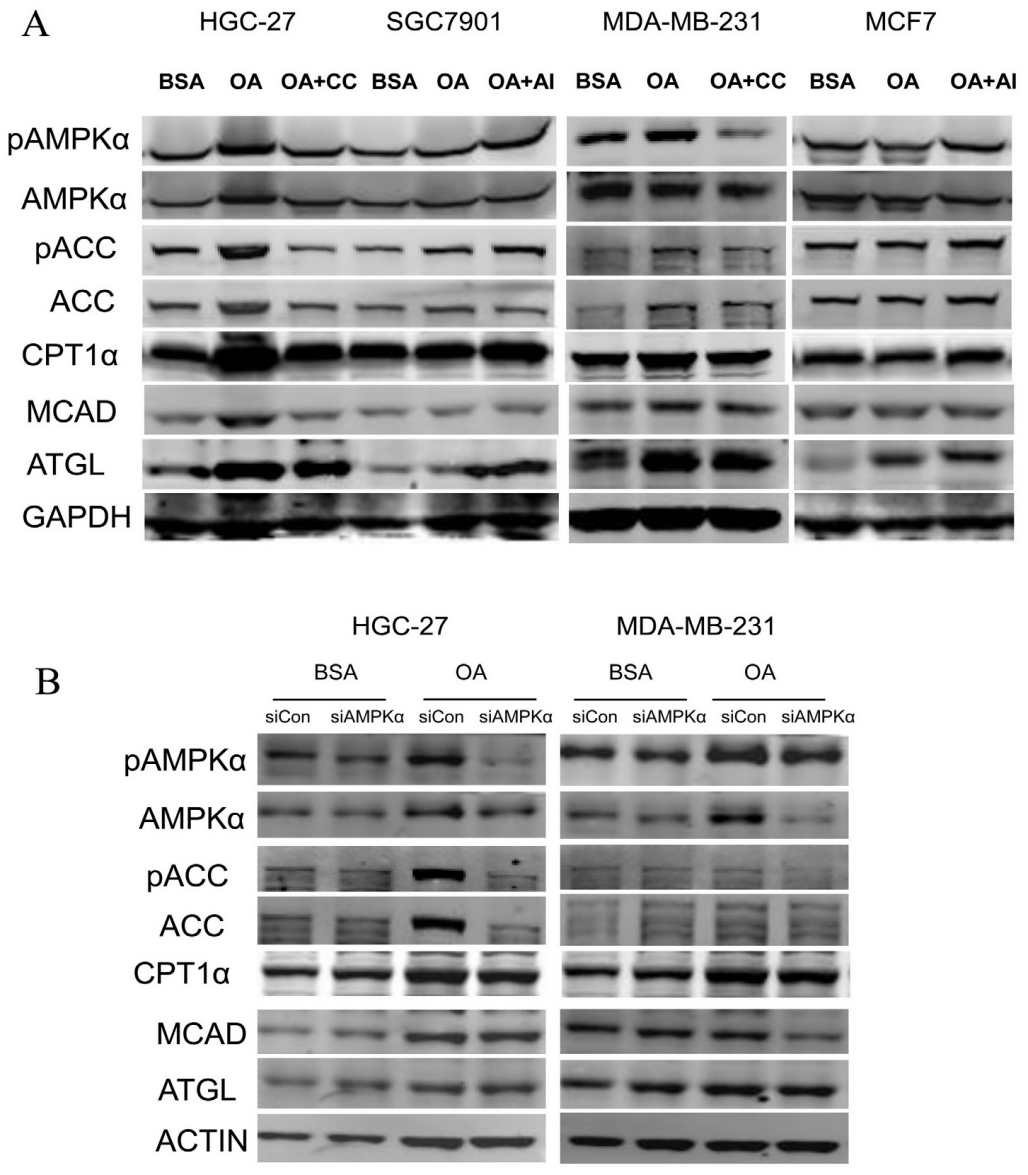
AMPK is involved in the OA-mediated protein expressions of fatty acid metabolism enzymes. The gastric carcinoma cells and breast cancer cells were cultured with 0.5% BSA or 400 µM of BSA-bound oleic acid with 5 µM of Compound C or with 100 µM of AICAR for 48 h. The levels of pAMPKα (Thr172), the total AMPKα, pACC (Ser79), the total ACC, CPT1α, MCAD and ATGL were determined by western blot analysis using 50 µg of the total proteins from each sample. A representative blot is shown. (B) The cells transfected with control-siRNA or AMPKα1-siRNA (24 hours) were treated with 0.5% BSA or 400 µM of BSA-bound oleic acid for another 48 hours. The expressions of pAMPKα (Thr172), the total AMPKα, pACC (Ser79), the total ACC, CPT1α, MCAD and ATGL were determined by western blot analysis.

To understand the accurate molecular mechanism by which AMPK activation determined the effects of OA on cellular function, we examined the status of AMPK activation and the expression of related enzymes regarding the FFA metabolism. It was parallel to the data shown in Figure 3 and demonstrated that OA activated the AMPK signalling by up-regulating the phosphorylation of AMPKα and ACC. This up regulation is accompanied by an increase in the expression of CPT1α, whereas this induction was obviously attenuated by Compound C in the HGC-27 and MDA-MB-231 cells. Apparently, AMPK was not stimulated in the MCF-7 or SGC7901 cells in the presence of OA. When AICAR was added to the medium, the level of the phosphorylated AMPKα and ACCwas increased, as well as the CPT1α and MCAD levels, which catalysed crucial steps in the mitochondrial fatty acid oxidation (Fig. 6A). To confirm further the implication of the AMPK signalling in the OA action, we transfected the AMPKα1-siRNA in the HGC-27 and MAD-MB-231 cells, and then treated the cells with OA or BSA, as the control. As shown in Figure 6B, silencing the AMPKα caused a reduction in the expression of phosphorylated AMPKα and ACC with CPT1α and MCAD.

Although the expression of ATGL was completely stimulated in all the cells treated by OA compared to the control cells (Fig. 3A), it was not influenced by the AMPK status (Fig. 6A, 6B), suggesting that ATGL, at least at the protein level, was not regulated by AMPK in the conditions of the OA treatment.

These data suggested that AMPK is critical for OA stimulation of cancer cell survival and growth through the promotion of fatty acid oxidation.

## Discussion

The data presented in this study demonstrated that oleic acid selectively promotes cell proliferation and migration in high metastatic cancer cells whereas it has inhibitory effects in low metastatic cancer cells. Although for decades it has been well accepted that AMPK activation suppresses cell growth and proliferation [29], [30], new evidence that AMPK activation promotes certain human cancer cell growth and survival emerged recently in such as prostate and ovarian cancer and in glioma cells [23], [30], [31]. Our data reinforced this new concept with additional information elucidated the unique characteristics of energy metabolism in high metastatic cancer cells encountering metabolic stress from oleic acid exposure, which was mediated by AMPK activation that resulted in enhanced fatty acid oxidation.

The physiological roles of oleic acid in health and disease in humans are minimally investigated and understood. High-metastatic cancer cells and low metastatic cancer cells had opposite responses to oleic acid treatment with respect to cell survival and migration. As shown, the HGC-27 and MDA-MB-231 cells grew faster in the presence of oleic acid (Fig. 1&2). There is increasing evidence that cancer cells show specific alterations in different aspects of lipid metabolism. The changes in lipid metabolism affect numerous cellular processes, including cell growth, proliferation and motility [32]. Metabolically, the high metastastic cancer cells setting from low metastastic cancer cells are endowed with rapid growth and high migration ability, which are because of the distinct metabolism trait. We concluded that high metastatic cancer cells with high-level consumption for energy utilise OA more efficiently than their counterpart cells.

The molecular differences induced by OA in these two cell groups are unclear. AMPK is a key regulator of energy homeostasis within cells. On activation, AMPK switches off the ATP-consuming biosynthetic pathways (e.g., fatty acid synthesis) and activates the ATP-generating metabolic pathways (e.g., fatty acid oxidation) to preserve the ATP levels for cell survival [33]. This study was the first to unmask AMPK activation as a possible mechanism for the intrinsic correlation between OA and different cell fates. Mechanically, we found increased AMPK activation, based on Thr172 phosphorylation, in the high metastatic cancer cells exposed to OA, which exhibited the promotion of cell proliferation and migration. As shown, the total and phosphorylated AMPKα and ACC amounts as well as CPT1α were remarkably high in the HGC-27 and MDA-MB-231 cells, although the ATGL level was up-regulated in these two cell groups (Fig. 3). The role of fatty acid oxidation in the altered energy metabolism of cancer cells is less clear and might have been influenced by the culture conditions to which the cells were exposed. Our data suggested that β-oxidation rather than lipolysis is the main change in the microenvironment with OA; the data support a possible functional link between AMPK and the altered energy metabolism in cancer cells. The selective AMPK activation in high metastatic cancer cells results in energy metabolism plasticity or adaptation. This assumption was further sustained by the data obtained by inhibition or stimulation of AMPK activity. As shown in Figure 4, in the HGC-27 and MDA-MB-231 cells with AMPK activation induced by OA, the inhibition of AMPK by Compound C or AMPKα1 siRNA attenuated the increase of cell growth and migration, and the artificial activation of AMPK by AICAR in the SGC7901 and MCF7 cells compensated these inhibitory effects induced by OA.

AMPK contributes to the maintenance of high ATP levels, which might remain remarkably stable for high energy-dependent molecular activities. Consistently, we found elevated levels of cellular ATP with AMPK activation and reduced ATP levels with inhibition of AMPK by Compound C in the OA-treated HGC-27 and MDA-MB-231 cells, whereas AIACR activated AMPK, subsequently leading to the ATP production in the SGC7901 and MCF7 cells (Fig. 5E). The significant increase in oxygen consumption induced by oleic acid could be reduced by AMPK inhibition (Fig. 5C&D). ATP production apparently results from the activation of AMPK and downstream enzymes. To support this mechanism, the study interpreted the intrinsic link between AMPK activation and the cellular behaviours. As shown in Figure 6, Compound C or AMPKα1 siRNA reduced the total and phosphorylated AMPKα and ACC as well as the subsequent CPT1α and MCAD levels, and AIACR compensated for these corresponding changes. One possible explanation for the difference between high and low malignancy cells would be the inability of the low malignancy cells to increase AMPKα and ACC gene expression in response to OA.Thus, it is possible that the long-term effects promoted by the constitutive AMPK activation support the adaptation of metastatic cells to the energy pathways that are predominant in specific metabolic stress and that contribute to the growth advantage of tumour cells.

The two AMPKα variants (AMPK-α1 and AMPK-α2) have a differential localisation pattern in mammalian cells, with the AMPK-α1 subunit being localised in the cytoplasm whereas the AMPK-α2 subunit is localised in the nucleus [34]. The transfection of cells with AMPK-α1 siRNA, and not AMPK-α2siRNA, abolished the effects of OA on the cell viability and protein expressions of phosphorylated ACC, CPT1α and MCAD. The downstream genes of AMPK, including ACC, that are localised in the cytoplasm suggest that at least some of the effects that were observed in the study are primarily because of the alpha-1 activity.

The AMPK system is activated by a large variety of cellular stresses that deplete ATP, such as glucose deprivation, ischemia, hypoxia, oxidative stress and hyperosmotic stress.It has been demonstrated in cultured adipocytes that agents that increase intracellular cAMP increase the activity of AMPK [35]. Wu has reported that treatment of bovine aortic endothelial cells (BAECs) with OA-NO2 induced a significant increase in AMPKThr172 phosphorylation and AMPK activity [36]. Recent reports have shown that oncogenic Ras, MYC and *Pten* deletion activates AMPK and that this activation stimulates cell proliferation [31], [37]. We hypothesise that the intracellular stresses or the oncogene induced by OA treatment in high metastatic cancer cells contribute to the activation of AMPK. The accurate mechanisms involved inthe regulation of AMPK in response to OA warrant further investigation.

Oleic acid prompted cell proliferation and migration in high metastatic cancer cells via enhanced β-oxidation mediated by AMPK activation. Provided that these observations might be extended to the in vivo situation, it could be postulated that a microenvironment rich in oleic acid might favour tumour progression. We reported for the first time that activated AMPK is involved in OA-induced cell proliferation and migration in terms of energy metabolism, which offers novel potential targets for the chemoprevention of human cancer.

## Supporting Information

Figure S1 **Effects of oleic acid on cell apotosis in various human cancer cell lines.** Human gastric cancer cell lines (HGC-27 and SGC7901), breast cancer cell lines (MDA-MB-231 and MCF-7) were starved for 12 hours, and then incubated with 0.5% BSA as control or 400 µM BSA-bound oleic acid for 48 hours. (A) Cells were stained with Hoechst 33258 and photographed (magnification 200x). (B) Cells were stained with AnnexinV and PI respectively and then quantified by flow cytometry analysis. Values are the mean ± SD, n = 3;#p<0.05 for OA compared with BSA.Student’s t test. (C) Caspase 3 and Caspase 9 were examined by Western blotting analysis with actin loaded as a control.
(TIF)

Figure S2 **The oxygen consumption rate in MDA-MB-231 and MCF-7 cells treated with OA.** Cells were incubated with 0.5% BSA as control or 400 µM BSA-bound oleic acid for 48 hours. Five million cells were resuspended in 1 ml of fresh warm mediumpre-equilibrated with 21% oxygen and the oxygen content in thecell suspension medium was constantly monitored for 10 min andoxygen consumption rate was recorded. Values are the mean ± SD, n = 3;*p<0.05 for OA compared with BSA.Student’s t test.
(TIF)

Figure S3 **Representative bolt of pAMPK and AMPK in control-siRNA and AMPKα1-siRNA cells.** MDA-MB-231 and HGC-27 cells at approximately 60% confluency were transfected with control-siRNA and AMPKα1-siRNA using Lipofectamine 2000. Transfections were performed in serum-free medium for 8 hours. After incubation, transfection complexes were removed and replaced with serum-free medium. (A)The expressions of pAMPKα and AMPKα were determined by Western blotting analysis.(B)Quantification of Protein expression by densitometry from three independent experiments,normalised to actin. Values are expressed as percent of control cells, given as mean ± SD, n = 3; *p<0.05 for AMPKα1-siRNAcompared with control-siRNA.Student’s t test.
(TIF)

Figure S4 **Oil red O staining in cells treated with OA in the presence of Compound C or AICAR.** HGC-27 and SGC7901 cells were cultured with 0.5% BSA or 400 µM BSA-bound oleic acid either with 5 µM Compound C or with 100 µM AICAR. Cells were stained with oil red O and photographed (200× magnification).
(TIF)
