# Supplementary figures and images for: High Metastaticgastric and Breast Cancer Cells Consume Oleic Acid in an AMPK Dependent Manner
Source: PLoS One. 2014 May 13;9(5):e97330. doi: 10.1371/journal.pone.0097330 (PMC4019637; doi:10.1371/journal.pone.0097330)

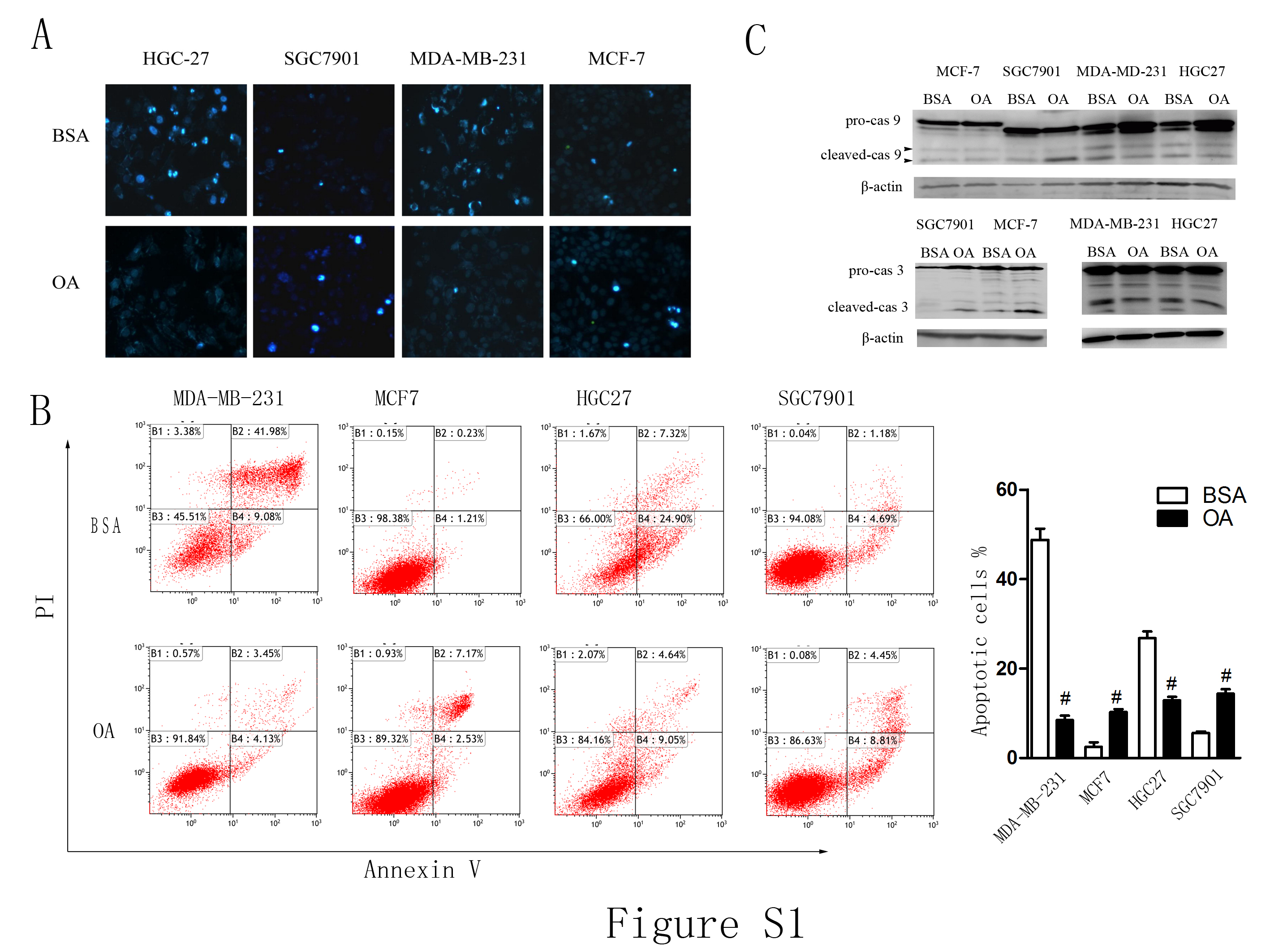

Supplement: Figure S1 — Effects of oleic acid on cell apotosis in various human cancer cell lines. Human gastric cancer cell lines (HGC-27 and SGC7901), breast cancer cell lines (MDA-MB-231 and MCF-7) were starved for 12 hours, and then incubated with 0.5% BSA as control or 400 µM BSA-bound oleic acid for 48 hours. (A) Cells were stained with Hoechst 33258 and photographed (magnification 200x). (B) Cells were stained with AnnexinV and PI respectively and then quantified by flow cytometry analysis. Values are the mean ± SD, n = 3;#p<0.05 for OA compared with BSA.Student’s t test. (C) Caspase 3 and Caspase 9 were examined by Western blotting analysis with actin loaded as a control. (TIF) [file pone.0097330.s001.tif]

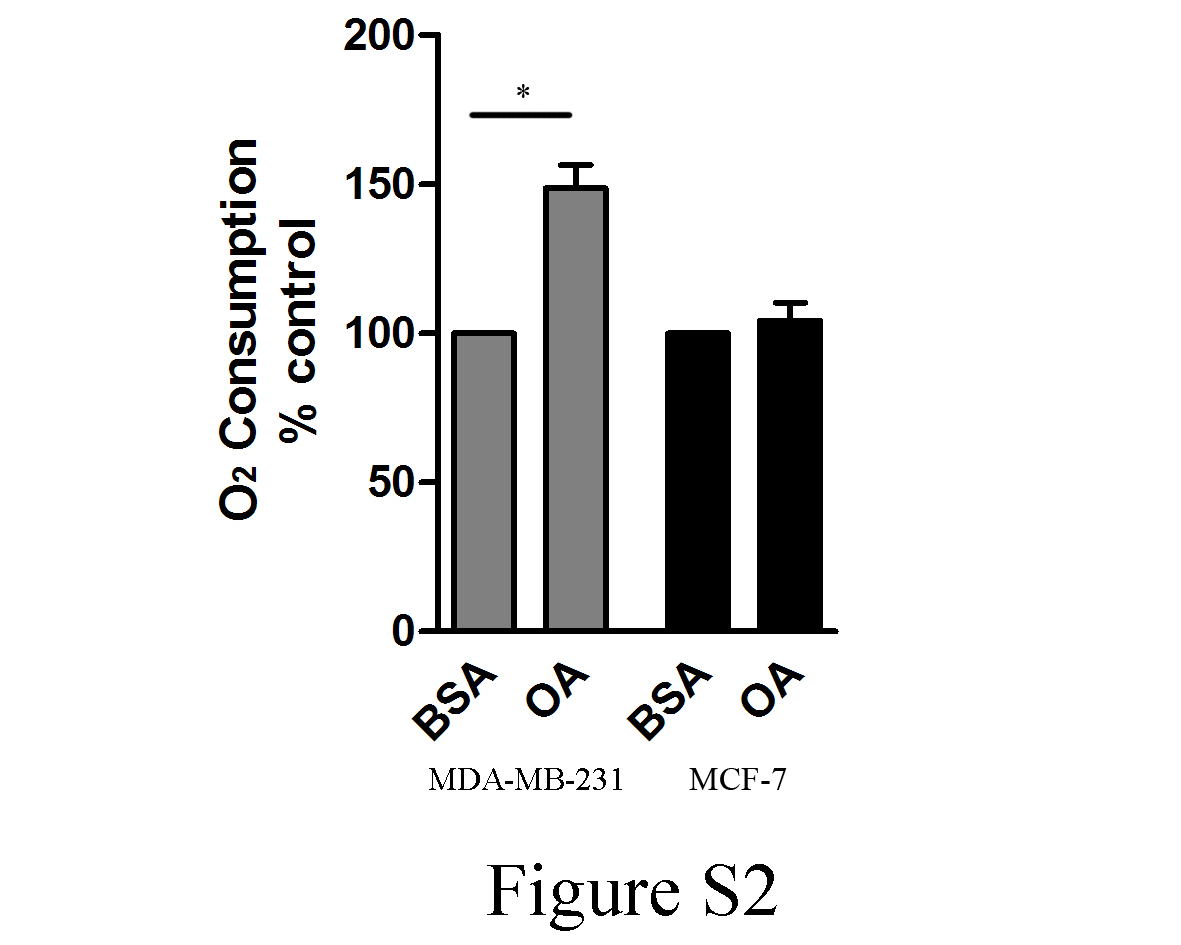

Supplement: Figure S2 — The oxygen consumption rate in MDA-MB-231 and MCF-7 cells treated with OA. Cells were incubated with 0.5% BSA as control or 400 µM BSA-bound oleic acid for 48 hours. Five million cells were resuspended in 1 ml of fresh warm mediumpre-equilibrated with 21% oxygen and the oxygen content in thecell suspension medium was constantly monitored for 10 min andoxygen consumption rate was recorded. Values are the mean ± SD, n = 3;*p<0.05 for OA compared with BSA.Student’s t test. (TIF) [file pone.0097330.s002.tif]

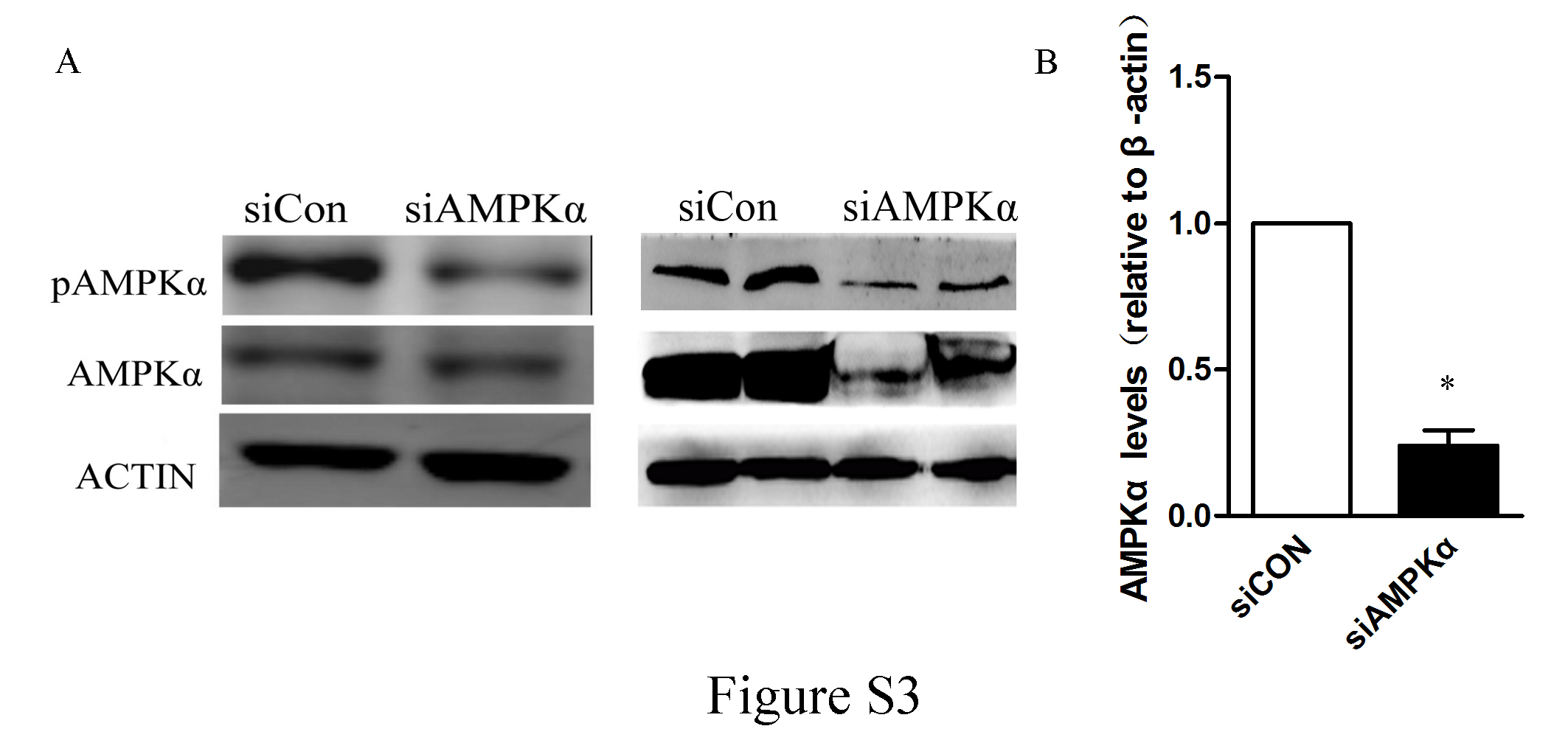

Supplement: Figure S3 — Representative bolt of pAMPK and AMPK in control-siRNA and AMPKα1-siRNA cells. MDA-MB-231 and HGC-27 cells at approximately 60% confluency were transfected with control-siRNA and AMPKα1-siRNA using Lipofectamine 2000. Transfections were performed in serum-free medium for 8 hours. After incubation, transfection complexes were removed and replaced with serum-free medium. (A)The expressions of pAMPKα and AMPKα were determined by Western blotting analysis.(B)Quantification of Protein expression by densitometry from three independent experiments,normalised to actin. Values are expressed as percent of control cells, given as mean ± SD, n = 3; *p<0.05 for AMPKα1-siRNAcompared with control-siRNA.Student’s t test. (TIF) [file pone.0097330.s003.tif]

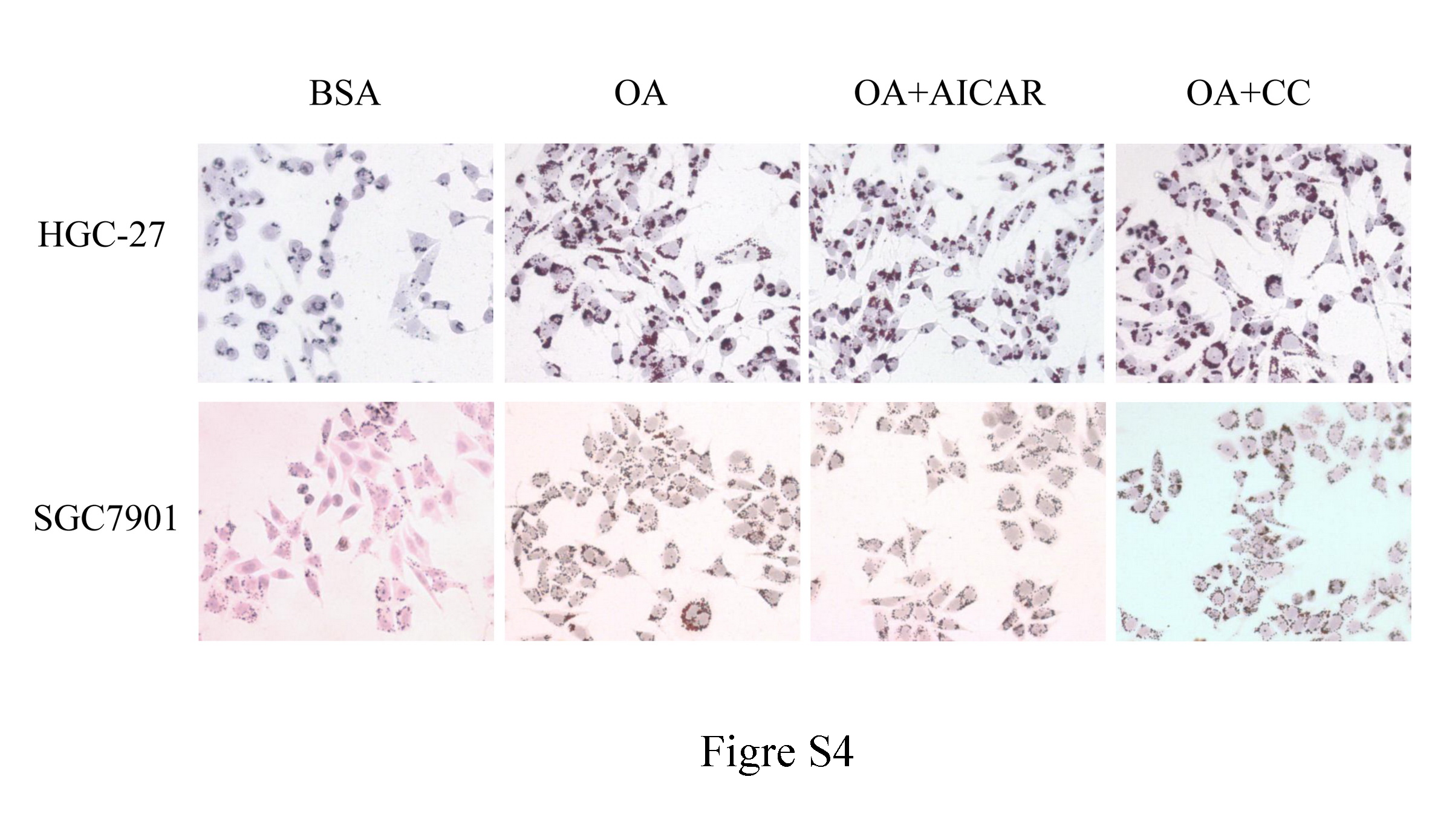

Supplement: Figure S4 — Oil red O staining in cells treated with OA in the presence of Compound C or AICAR. HGC-27 and SGC7901 cells were cultured with 0.5% BSA or 400 µM BSA-bound oleic acid either with 5 µM Compound C or with 100 µM AICAR. Cells were stained with oil red O and photographed (200× magnification). (TIF) [file pone.0097330.s004.tif]
